# Assessment of Incidence and Factors Associated With Severe Maternal Morbidity After Delivery Discharge Among Women in the US

**DOI:** 10.1001/jamanetworkopen.2020.36148

**Published:** 2021-02-02

**Authors:** Jiajia Chen, Shanna Cox, Elena V. Kuklina, Cynthia Ferre, Wanda Barfield, Rui Li

**Affiliations:** 1 Division of Reproductive Health, National Center for Chronic Disease Prevention and Health Promotion, Centers for Disease Control and Prevention, Atlanta, Georgia; 2 Now with Division of Research, Office of Epidemiology and Research, Maternal and Child Health Bureau, Health Resources and Services Administration, Rockville, Maryland

## Abstract

**Importance:**

Previous efforts to examine severe maternal morbidity (SMM) in the US have focused on delivery hospitalizations. Little is known about de novo SMM that occurs after delivery discharge.

**Objective:**

To investigate the incidence, timing, factors, and maternal characteristics associated with de novo SMM after delivery discharge among women in the US.

**Design, Setting, and Participants:**

In this retrospective cohort study, data from the IBM MarketScan Multi-State Medicaid database and the IBM MarketScan Commercial Claims and Encounters database were used to construct a sample of women aged 15 to 44 years who delivered between January 1, 2010, and September 30, 2014. Severe maternal morbidity was reported by the timing of diagnosis, and the associated maternal characteristics were examined. Women in the Medicaid and commercial insurance sample were classified into 3 distinct outcome groups: (1) those without any SMM during the delivery hospitalization and the postdelivery period (reference group), (2) those who exhibited at least 1 factor associated with SMM during the delivery hospitalization, and (3) those who exhibited any factor associated with de novo SMM after delivery discharge (defined as SMM that was first diagnosed in the inpatient setting during the 6 weeks [or 42 days] after discharge from the delivery hospitalization, conditional on no factor associated with SMM being identified during delivery). Data were analyzed from February to July 2020.

**Exposures:**

Timing of SMM diagnosis.

**Main Outcomes and Measures:**

Women with SMM were identified using diagnosis and procedure codes from the *International Classification of Diseases, Ninth Revision, Clinical Modification* for the 21 factors associated with SMM that were developed by the Centers for Disease Control and Prevention.

**Results:**

A total of 2 667 325 women in the US with delivery hospitalizations between 2010 and 2014 were identified; of those, 809 377 women (30.3%) had Medicaid insurance (30.3%; mean [SD] age, 25.6 [5.5] years; 51.1% White), and 1 857 948 women (69.7%; mean [SD] age, 30.6 [5.4] years; 36.4% from the southern region of the US) had commercial insurance. Among those with Medicaid insurance, 17 584 women (2.2%) experienced SMM during the delivery hospitalization, and 3265 women (0.4%) experienced de novo SMM after delivery discharge. Among those with commercial insurance, 32 079 women (1.7%) experienced SMM during the delivery hospitalization, and 5275 women (0.3%) experienced de novo SMM after hospital discharge. A total of 5275 SMM cases (14.1%) and 3265 SMM cases (15.7%) among women with commercial and Medicaid insurance, respectively, developed de novo within 6 weeks after hospital discharge; of those, 3993 cases (75.7%) in the commercial insurance cohort and 2399 cases (73.5%) in the Medicaid cohort were identified in the first 2 weeks after discharge. The most common factors associated with SMM varied based on the timing of diagnosis. In the Medicaid population, non-Hispanic Black women (adjusted odds ratio [aOR], 1.53; 95% CI, 1.48-1.58), Hispanic women (aOR, 1.46; 95% CI, 1.37-1.57), and women of other races or ethnicities (aOR, 1.40; 95% CI, 1.33-1.47) had higher rates of SMM during delivery hospitalization than non-Hispanic White women; however, only the disparity between Black and White women (aOR, 1.69; 95% CI, 1.57-1.81) persisted into the postdischarge period.

**Conclusions and Relevance:**

In this study, 14.1% of SMM cases in the Medicaid cohort and 15.7% of SMM cases in the commercial insurance cohort first occurred after the delivery hospitalization, with notable disparities in factors and maternal characteristics associated with the development of SMM. These findings suggest a need to expand the focus of SMM assessment to the postdelivery discharge period.

## Key Points

**Question:**

What proportion of de novo severe maternal morbidity is diagnosed after delivery discharge, and what are the most common factors and maternal characteristics associated with severe maternal morbidity among women in the US?

**Findings:**

In this cohort study of 2 667 325 women in the US with delivery hospitalizations between 2010 and 2014, 14% and 16% of severe maternal morbidity among those with commercial and Medicaid insurance, respectively, developed de novo within 6 weeks after delivery discharge. The most common factors and maternal characteristics associated with severe maternal morbidity after delivery were different than those identified at delivery.

**Meaning:**

The study’s findings suggest that expanding the focus of severe maternal morbidity assessment to the postdelivery discharge period could improve understanding of severe maternal morbidity and may create opportunities to improve maternity care.

## Teaser

This cohort study assesses the incidence, timing, factors, and maternal characteristics associated with de novo severe maternal morbidity after delivery discharge among women in the US.

## Introduction

Each year, approximately 700 women in the US die of complications associated with pregnancy and childbirth.^1^ Among all pregnancy-associated deaths, a sizeable proportion occur in the postpartum period. For example, a recent report using data from the Centers for Disease Control and Prevention Pregnancy Mortality Surveillance System found that 18.6% of maternal deaths occurred on days 1 to 6 postpartum, and 21.4% of maternal deaths occurred on days 7 to 42 postpartum.^2^ Moreover, the underlying factors associated with death varied by the timing of these deaths.^2,3,4^

For every maternal death, it is estimated that 50 to 100 women experience severe maternal morbidity (SMM), which is defined as life-threatening complications without timely identification and proper management that may be associated with deaths.^5,6,7^ The rates of many factors associated with SMM have increased in the past few decades.^6,8^ The recorded SMM rate increased by 75%, from 73.8 cases per 100 000 hospitalizations between 1998 and 1999 to 129.1 cases per 100 000 hospitalizations between 2008 and 2009, and the rate has remained high (approximately 140.0 cases per 100 000 hospitalizations in 2014).^6,9^ Factors such as increases in maternal age and preexisting chronic conditions have been documented as potential associations.^10,11^

Recognizing the increasing prevalence of SMM and its association with maternal death, policy and research efforts have continued to be aimed at understanding the occurrence and prevention of SMM.^7,12^ However, previous studies of SMM in the US have primarily focused on severe complications during the delivery hospitalization because of limitations in available data.^5,6,13,14,15^ A few exceptions are noted in studies by Callaghan et al,^6^ Harvey et al,^16^ and Girsen et al.^17^ Nonetheless, little is known about the rate and pattern of de novo postpartum SMM because these studies did not explicitly examine such cases. Similar to pregnancy-associated mortality, the distribution and characteristics of SMM that is first diagnosed during the delivery hospitalization may differ from those of SMM that first occurs after delivery discharge.^2,3,4^

The study’s objective was to assess the incidence, timing, factors, and maternal characteristics associated with de novo SMM after delivery discharge compared with SMM that occurred during delivery hospitalization using a sample of women covered by commercial insurance and Medicaid. We specifically examined the timing of SMM after delivery discharge, compared the factors associated with postdelivery SMM with those observed during the delivery hospitalization, and investigated the associations between selected maternal characteristics and SMM that was identified during delivery and after delivery.

## Methods

This study was approved by the Centers for Disease Control and Prevention and deemed exempt from informed consent because individuals could not be identified in the data set. The study followed the Strengthening the Reporting of Observational Studies in Epidemiology (STROBE) guideline for cohort studies.

We used the IBM MarketScan Multi-State Medicaid database and the IBM MarketScan Commercial Claims and Encounters database to conduct a retrospective cohort study using a sample of women aged 15 to 44 years who delivered between January 1, 2010, and September 30, 2014. The Medicaid database contains health care claims for more than 6 million Medicaid enrollees in 9 to 13 states each year. The commercial claims database comprises a nationwide convenience sample of health insurance claims among individuals with commercial insurance and includes adjudicated claims for more than 30 million employees and their dependents. Both data sets include patient-level data on enrollment, inpatient and outpatient services, and outpatient drug use. Patients were assigned a unique identifier, allowing us to develop a sample of patients with data through pregnancy and the postpartum period. In general, the data structure and variables in the 2 data sets were similar, although the availability of patient characteristics is not uniform in the 2 databases. For example, the Medicaid database includes patient race and ethnicity but not geographic location (eg, state or census region) of residence, whereas the opposite information is available in the commercial claims database.

Our analytic sample consisted of women aged 15 to 44 years who had a live birth or stillbirth delivery during the observation period. The women were identified based on an algorithm that was previously developed using claims data.^18^ A woman may have had more than 1 delivery during our study period. We randomly selected 1 delivery for women with multiple deliveries to avoid within-individual association. We restricted our sample to those with continuous insurance enrollment for at least 6 weeks after the discharge date of the delivery hospitalization. We considered the period of 6 weeks to approximate the standard for maternal mortality used by the World Health Organization and the National Center for Health Statistics of the Centers for Disease Control and Prevention.^19^ Because the exact date of delivery was not available, we used the discharge date of the delivery hospitalization as the beginning date of the 6-week period.

We used the 21 factors associated with SMM that were developed by the Centers for Disease Control and Prevention to identify women with at least 1 factor associated with SMM during delivery and within the first 6 weeks after delivery discharge. Severe maternal morbidity was identified using diagnosis and procedure codes from the *International Classification of Diseases, Ninth Revision, Clinical Modification* (*ICD-9-CM*) (eTable 1 in the Supplement). Each woman in the sample was classified into 3 distinct outcome groups: (1) those without any SMM during the delivery hospitalization and the postdelivery period (reference group), (2) those who exhibited at least 1 factor associated with SMM during the delivery hospitalization, and (3) those who exhibited any factor associated with de novo SMM after delivery discharge. For each delivery selected in the study, we defined de novo SMM after discharge as SMM that was first diagnosed in the inpatient setting during the 6 weeks (or 42 days) after discharge from the delivery hospitalization, conditional on no factor associated with SMM being identified during delivery.

To ensure that we identified true de novo SMM cases, we excluded women who experienced any SMM event in the 2 months before delivery hospitalization (1043 women in the Medicaid cohort and 1186 women in the commercial insurance cohort). Some of the women excluded also experienced an SMM event during and after the delivery hospitalization (eTable 2 in the Supplement). We also tested restriction periods of 4 months and 0 days, neither of which had consequences for our main conclusions.

### Statistical Analysis

We examined the timing of de novo SMM after delivery discharge using data from the Medicaid and commercial claims databases. We compared the distribution of the 3 groups of women in the analytical sample by database (Medicaid vs commercial) using unpaired 2-tailed Pearson χ^2^ tests. Within each database sample, we also examined the distribution of the factors associated with SMM that were identified during the 2 periods (ie, during and after delivery hospitalization). One of the factors, blood transfusion, represented a substantial event; however, transfusion may not always reflect SMM in the absence of other factors.^20^ Therefore, we excluded blood transfusion when examining the distribution of factors associated with SMM.

To explore whether associations between maternal characteristics and SMM differed by the timing of SMM events, we constructed a categorical variable indicating various SMM statuses for each woman following the classification methods discussed in the previous paragraphs. Each woman had 1 of the 3 distinct outcomes, with the first category (reference group) being no SMM during or after the delivery hospitalization, the second category being any SMM during the delivery hospitalization, and the third category being any de novo SMM after the delivery discharge.

To ensure our results were comparable with previous research, the second group (any SMM during the delivery hospitalization) also included a small percentage of women who experienced SMM after discharge (eTable 2 in the Supplement). Most previous research on SMM has examined any SMM during delivery hospitalization vs no SMM. By creating an additional category, we were able to fit a multinomial logistic regression model to simultaneously analyze (1) any SMM during delivery hospitalization vs no SMM and (2) any de novo SMM after discharge vs no SMM.

The regression model examined the association between SMM status and maternal demographic characteristics (age, race, and ethnicity for patients with Medicaid insurance and area of residence for patients with commercial insurance). We also included other maternal characteristics, such as delivery outcome (live birth vs stillbirth) and delivery method (vaginal vs cesarean), based on information available in the delivery hospitalization records (eTable 3 in the Supplement). Multinomial regression analysis allowed comparison of the coefficients of the same maternal characteristic (eg, race) for the presence of SMM at different periods. We conducted the regression analyses using Stata software, version 14 (StataCorp).^21^ The analyses were not weighted. Data were analyzed from February to July 2020.

## Results

Among 2 667 325 women in the US with delivery hospitalizations between 2010 and 2014, 809 377 women (30.3%; mean [SD] age, 25.6 [5.5] years; 51.1% White, 33.2% Black, 5.1% Hispanic, and 10.6% of other race or ethnicity [other races and ethnicities not specified in the database]) had Medicaid coverage and 1 857 948 women (69.7%; mean [SD] age, 30.6 [5.4] years; 17.1% from the northeastern region, 24.0% from the north central region, 36.4% from the southern region, and 20.6% from the western region of the US) had commercial insurance (Table 1 and Table 2). In total, 17 584 women (2.2%; 95% CI, 2.1%-2.2%) in the Medicaid cohort and 32 079 women (1.7%; 95% CI, 1.7%-1.8%) in the commercial insurance cohort had at least 1 factor associated with SMM that was identified during the delivery hospitalization. A total of 3265 women (0.4%; 95% CI, 0.4%-0.4%) in the Medicaid cohort and 5275 women (0.3%; 95% CI, 0.3%-0.3%) in the commercial insurance cohort were diagnosed with de novo SMM after delivery discharge (Table 1). De novo SMM cases after discharge represented 15.7% (95% CI, 15.1%-16.2%) of total SMM cases in the Medicaid cohort and 14.1% (95% CI, 13.8%-14.5%) of total SMM cases in the commercial insurance cohort (Table 1).

**Table 1.** SMM Diagnosis by Timing of Diagnosis and Insurance Type, 2010 to 2014^a^

Timing of diagnosis	Insurance				Total	
	Medicaid		Commercial			
	No. (%)	95% CI, %	No. (%)	95% CI, %	No. (%)	95% CI, %
**All women**						
Total participants, No.	809 377	NA	1 857 948	NA	2 667 325	NA
No SMM during and after delivery hospitalization	788 528 (97.4)	97.4-97.5	1 820 594 (98.0)	98.0-98.0	2 609 122 (97.8)	97.8-97.8
Any SMM during delivery hospitalization	17 584 (2.2)	2.1-2.2	32 079 (1.7)	1.7-1.8	49 663 (1.9)	1.8-1.9
Any de novo SMM during postpartum hospitalization	3265 (0.4)	0.4-0.4	5275 (0.3)	0.3-0.3	8540 (0.3)	0.3-0.3
**Women with SMM**						
Total participants, No.	20 849	NA	37 354	NA	NA	NA
Any SMM during delivery hospitalization	17 584 (84.3)	83.8-84.8	32 079 (85.9)	85.5-86.2		
Any de novo SMM during postpartum hospitalization	3265 (15.7)	15.1-16.2	5275 (14.1)	13.8-14.5		

Abbreviations: NA, not applicable; SMM, severe maternal morbidity.

^a^ Data were obtained from the 2010 to 2014 IBM MarketScan Multi-State Medicaid and IBM MarketScan Commercial Claims and Encounters databases. Pearson χ^2^ test for the equal distribution of different SMM status across 2 populations: χ^2^ = 871.99; *P* < .001.

**Table 2.** Maternal Characteristics by Insurance Type and Timing of Diagnosis, 2010 to 2014

Characteristic by insurance type	No. (%)			
	No SMM during and after delivery hospitalization	Any SMM during delivery hospitalization	Any de novo SMM during postpartum hospitalization	Total
**Medicaid**				
Total participants, No.	788 528	17 584	3265	809 377
Age group, y				
15-24	386 384 (49.0)	8210 (46.7)	1271 (38.9)	395 865 (48.9)
25-34	341 399 (43.3)	7447 (42.4)	1580 (48.4)	350 426 (43.3)
35-44	60 745 (7.7)	1927 (11.0)	414 (12.7)	63 086 (7.8)
Race/ethnicity				
Non-Hispanic White	404 793 (51.3)	7185 (40.9)	1367 (41.9)	413 345 (51.1)
Non-Hispanic Black	259 532 (32.9)	7373 (41.9)	1507 (46.2)	268 412 (33.2)
Hispanic	40 471 (5.1)	973 (5.5)	115 (3.5)	41 559 (5.1)
Other^a^	83 732 (10.6)	2053 (11.7)	276 (8.5)	86 061 (10.6)
Fetal outcome				
No stillbirth	780 804 (99.0)	16 936 (96.3)	3211 (98.3)	800 951 (99.0)
Any stillbirth	7724 (1.0)	648 (3.7)	54 (1.7)	8426 (1.0)
Delivery method				
Vaginal	544 187 (69.0)	6489 (36.9)	1638 (50.2)	552 314 (68.2)
Cesarean	244 341 (31.0)	11 095 (63.1)	1627 (49.8)	257 063 (31.8)
**Commercial**				
Total participants, No.	1 820 594	32 079	5275	1 857 948
Age group, y				
15-24	254 357 (14.0)	4221 (13.2)	722 (13.7)	259 300 (14.0)
25-34	1 131 666 (62.2)	18 409 (57.4)	2918 (55.3)	1 152 993 (62.1)
35-44	434 571 (23.9)	9449 (29.5)	1635 (31.0)	445 655 (24.0)
Census region				
Northeast	311 153 (17.1)	5639 (17.6)	800 (15.2)	317 592 (17.1)
North central	436 947 (24.0)	6635 (20.7)	1185 (22.5)	444 767 (23.9)
South	663 079 (36.4)	10 240 (31.9)	2207 (41.8)	675 526 (36.4)
West	372 119 (20.4)	8796 (27.4)	975 (18.5)	381 890 (20.6)
Unknown	37 296 (2.1)	769 (2.4)	108 (2.0)	38 173 (2.0)
MSA status				
MSA	1 553 440 (85.3)	27 477 (85.7)	4470 (84.7)	1 585 387 (85.3)
Non-MSA	230 320 (12.7)	3928 (12.2)	699 (13.3)	234 947 (12.6)
Unknown	36 834 (2.0)	674 (2.1)	106 (2.0)	37 614 (2.0)
Fetal outcome				
No stillbirth	1 806 700 (99.2)	31 324 (97.6)	5202 (98.6)	1 843 226 (99.2)
Any stillbirth	13 894 (0.8)	755 (2.4)	73 (1.4)	14 722 (0.8)
Delivery method				
Vaginal	1 178 674 (64.7)	12 640 (39.4)	2411 (45.7)	1 193 725 (64.2)
Cesarean	641 920 (35.3)	19 439 (60.6)	2864 (54.3)	664 223 (35.8)

Abbreviations: MSA, metropolitan statistical area (as defined by the US Office of Management and Budget); SMM, severe maternal morbidity.

^a^ Specific races included in this category were not available in the database.

**Figure 1.**
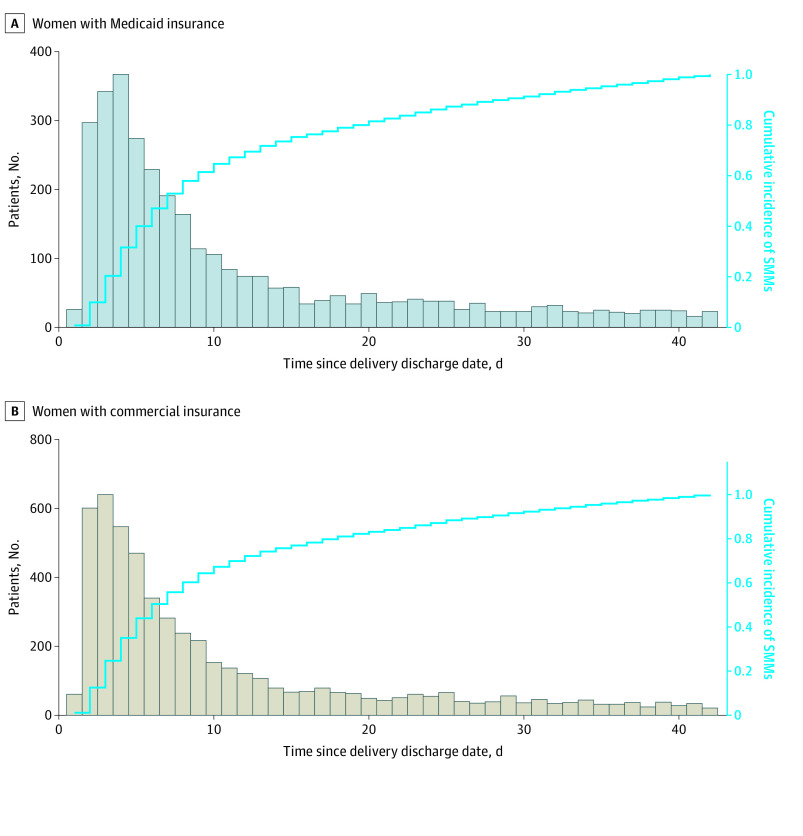
Timing of First Episode of De Novo Severe Maternal Morbidity Within 6 Weeks After Delivery Discharge, 2010 to 2014 A, Women with Medicaid insurance. B, Women with commercial insurance.

In an analysis of the timing (in days) of the first episode of SMM among women first diagnosed with SMM after delivery hospitalization, we found a similar pattern for women with Medicaid and commercial insurance (Figure 1). The number of SMM cases after delivery was highest within the first week after delivery hospitalization and decreased rapidly after the second week. A total of 2399 women (73.5%) in the Medicaid cohort and 3993 women (75.7%) in the commercial insurance cohort with de novo SMM after discharge were diagnosed in the first 2 weeks after discharge from the delivery hospitalization.

**Figure 2.**
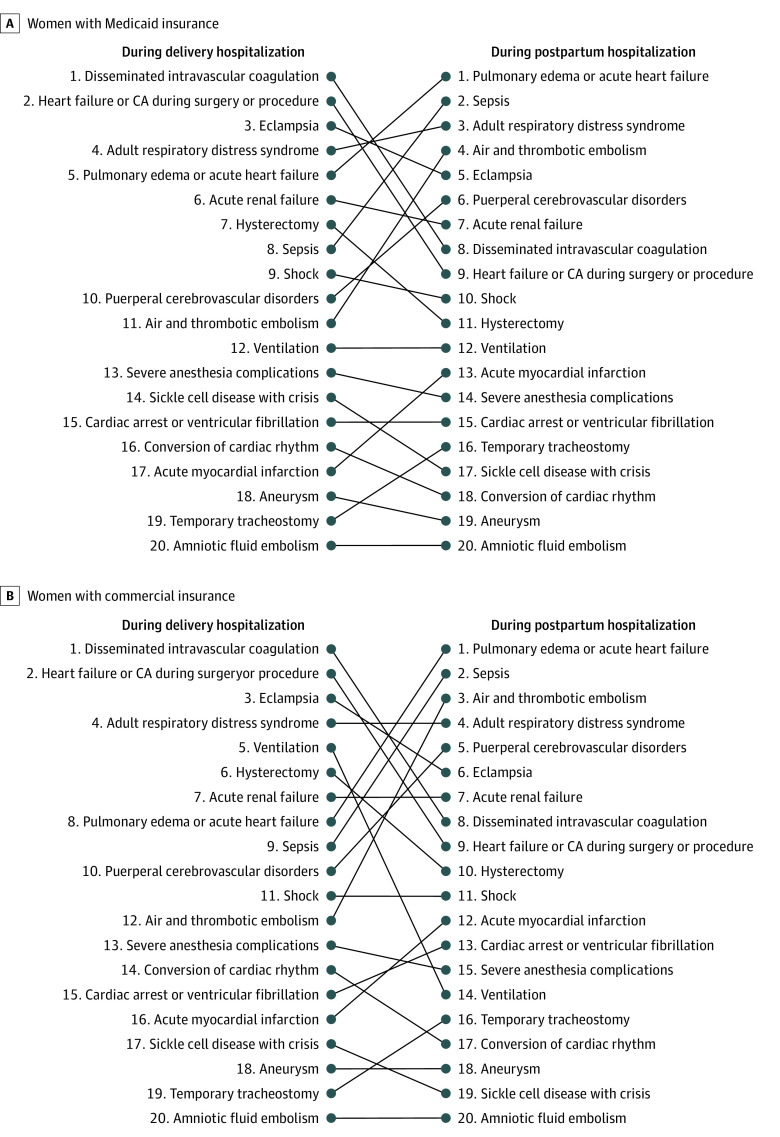
Relative Rank of Factors Associated With Severe Maternal Morbidity by Timing of Diagnosis, 2010 to 2014 Blood transfusion was excluded as a factor in the analysis. A, Women with Medicaid insurance. B, Women with commercial insurance.

The distributions of the factors associated with SMM were different among those diagnosed during delivery hospitalization compared with those diagnosed after delivery hospitalization; after excluding blood transfusion (*ICD-9-CM* code 99.0x), which was usually the dominant factor, 3 of the 5 most common factors associated with SMM during delivery no longer remained in the 5 most common factors associated with de novo SMM after discharge (Figure 2; eTable 4 in the Supplement). For example, in the Medicaid cohort, the 5 most common factors (excluding blood transfusion) during the delivery hospitalization were disseminated intravascular coagulation (*ICD-9-CM* codes 286.6, 286.9, and 666.3x), heart failure or cardiac arrest (*ICD-9-CM* codes 669.4 × and 997.1), eclampsia (*ICD-9-CM* code 642.6x), acute respiratory distress syndrome (*ICD-9-CM* codes 518.5x, 518.81, 518.82, 518.84, and 799.1), and pulmonary edema or acute heart failure (*ICD-9-CM* codes 518.4, 428.1, 428.0, 428.21, 428.23, 428.31, 428.33, 428.41, and 428.43). After delivery discharge, the 5 most common factors were pulmonary edema or acute heart failure, adult respiratory distress syndrome, sepsis (*ICD-9-CM* codes 038.xx, 995.91, 995.92, and 670.2x), air and thrombotic embolism (*ICD-9-CM* codes 415.1x, 673.0x, 673.2x, 673.3x, and 673.8x), and eclampsia.

In the Medicaid cohort, compared with women without SMM (n = 788 528), higher proportions of women with SMM in the delivery hospitalization period (n = 17 584) or postdelivery discharge period (n = 3265) were older (eg, for ages 35-44 years, 60 745 women [7.7%] without SMM vs 1927 women [11.0%] with SMM in the delivery period and 414 women [12.7%] with SMM in the postdelivery period), Black (259 532 women [32.9%] without SMM vs 7373 women [41.9%] with SMM in the delivery period and 1507 women [46.2%] with SMM in the postdelivery period), had a stillbirth delivery (7724 women [1.0%] without SMM vs 648 women [3.7%] with SMM in the delivery period and 54 women [1.7%] with SMM in the postdelivery period), or had a cesarean delivery (244 341 women [31.0%] without SMM vs 11 095 women [63.1%] with SMM in the delivery period and 1627 women [49.8%] with SMM in the postdelivery period) (Table 2). In the commercial insurance cohort, compared with women without SMM (n = 1 820 594), those with SMM during the delivery hospitalization period (n = 32 079) or postdelivery discharge period (n = 5275) also had higher proportions of older age groups (eg, for ages 35-44 years, 434 571 women [23.9%] without SMM vs 9449 women [29.5%] with SMM in the delivery period and 1635 women [31.0%] with SMM in the postdelivery period), stillbirth deliveries (13 894 women [0.8%] without SMM vs 755 women [2.4%] with SMM in the delivery period and 73 women [1.4%] with SMM in the postdelivery period), and cesarean deliveries (641 920 women [35.3%] without SMM vs 19 439 women [60.6%] with SMM in the delivery period and 2864 women [54.3%] with SMM in the postdelivery period).

For women in the Medicaid cohort, SMM that occurred during and after the delivery hospitalization was positively associated with older age (eg, for ages 35-44 years, adjusted odds ratio [aOR], 1.17 [95% CI, 1.11-1.23] in the delivery period and aOR, 1.85 [95% CI, 1.66-2.07] in the postdelivery period), non-White race (eg, for Black women, aOR, 1.53 [95% CI, 1.48-1.58] in the delivery period and aOR, 1.69 [95% CI, 1.57-1.81] in the postdelivery period), stillbirth delivery (aOR, 4.66 [95% CI, 4.29-5.07] in the delivery period and aOR, 1.73 [95% CI, 1.32-2.27] in the postdelivery period), and cesarean delivery (aOR, 3.88 [95% CI, 3.76-4.01] in the delivery period and aOR, 2.10 [95% CI, 1.95-2.25] in the postdelivery period) (Table 3). The extent of these associations varied by timing of diagnosis. For example, Black women (aOR, 1.53; 95% CI, 1.48-1.58), Hispanic women (aOR, 1.46; 95% CI, 1.37-1.57), and women of other races and ethnicities (aOR, 1.40; 95% CI, 1.33-1.47) had a higher likelihood of having any factor associated with SMM during delivery hospitalization compared with White women. However, only Black women (aOR, 1.69; 95% CI, 1.57-1.81) had a higher likelihood of experiencing SMM in the postdelivery discharge period compared with White women.

**Table 3.** Multinomial Logistic Regression Analysis of SMM for Selected Maternal Characteristics by Insurance Type and Timing of Diagnosis, 2010 to 2014^a^

Characteristic by insurance type	aOR (95% CI)		*P* value^b^
	Any factor vs no factor associated with SMM during delivery hospitalization	Any factor vs no factor associated with de novo SMM during postpartum hospitalization	
**Medicaid (n = 809 377)**^**c**^			
Age group, y			
15-24	1 [Reference]	1 [Reference]	NA
25-34	0.91 (0.88-0.94)	1.33 (1.24-1.44)	<.001
35-44	1.17 (1.11-1.23)	1.85 (1.66-2.07)	<.001
Race/ethnicity			
Non-Hispanic White	1 [Reference]	1 [Reference]	NA
Non-Hispanic Black	1.53 (1.48-1.58)	1.69 (1.57-1.81)	.02
Hispanic	1.46 (1.37-1.57)	0.86 (0.71-1.05)	<.001
Other^d^	1.40 (1.33-1.47)	0.96 (0.84-1.09)	<.001
Fetal outcome			
No stillbirth	1 [Reference]	1 [Reference]	NA
Any stillbirth	4.66 (4.29-5.07)	1.73 (1.32-2.27)	<.01
Delivery method			
Vaginal	1 [Reference]	1 [Reference]	NA
Cesarean	3.88 (3.76-4.01)	2.10 (1.95-2.25)	<.01
**Commercial (n = 1 857 948)**^e^			
Age group, y			
15-24	1 [Reference]	1 [Reference]	NA
25-34	0.89 (0.86-0.92)	0.86 (0.79-0.94)	.54
35-44	1.03 (1.00-1.07)	1.18 (1.08-1.29)	.008
Census region^f^			
Northeast	1 [Reference]	1 [Reference]	NA
North central	0.87 (0.84-0.91)	1.10 (1.01-1.21)	<.001
South	0.83 (0.80-0.85)	1.29 (1.18-1.39)	<.001
West	1.37 (1.32-1.41)	1.06 (0.97-1.16)	<.001
MSA status^f^			
MSA	1 [Reference]	1 [Reference]	NA
Non-MSA	1.04 (1.00-1.07)	1.06 (0.98-1.15)	.62
Fetal outcome			
No stillbirth	1 [Reference]	1 [Reference]	NA
Any stillbirth	3.80 (3.53-4.10)	2.01 (1.60-2.54)	<.001
Delivery method			
Vaginal	1 [Reference]	1 [Reference]	NA
Cesarean	2.89 (2.82-2.95)	2.13 (2.01-2.25)	<.001

Abbreviations: aOR, adjusted odds ratio; MSA, metropolitan statistical area (as defined by the US Office of Management and Budget); NA, not applicable; SMM, severe maternal morbidity.

^a^ Estimates are exponentiated coefficients from a single multinomial regression analysis for each insurance category. The dependent variable is a discrete variable with 3 distinct values: (1) no SMM during the delivery hospitalization and the postdelivery period (reference group), (2) at least 1 factor associated with SMM during the delivery hospitalization, and (3) any factor associated with de novo SMM after delivery discharge (defined as SMM that was first diagnosed in the inpatient setting during the 6 weeks [or 42 days] after discharge from the delivery hospitalization, conditional on no factor associated with SMM being identified during delivery).

^b^ *P* values were obtained from χ^2^ tests of the equality of coefficients.

^c^ Data were obtained from the 2010 to 2014 IBM MarketScan Multi-State Medicaid database.

^d^ Specific races included in this category were not available in the database.

^e^ Data were obtained from the 2010 to 2014 IBM MarketScan Commercial Claims and Encounters database.

^f^ Census region and MSA status are categorical variables with a missing/unknown category. Estimates for the missing/unknown category are not included in the table.

For women in the commercial insurance cohort, SMM identified during delivery hospitalization and after delivery discharge was positively associated with older age (eg, for ages 35-44 years, aOR, 1.03 [95% CI, 1.00-1.07] in the delivery period and aOR, 1.18 [95% CI, 1.08-1.29] in the postdelivery period), stillbirth (aOR, 3.80 [95% CI, 3.53-4.10] in the delivery period and aOR, 2.01 [95% CI, 1.60-2.54] in the postdelivery period), and cesarean delivery (aOR, 2.89 [95% CI, 2.82-2.95] in the delivery period and aOR, 2.13 [95% CI, 2.01-2.25] in the postdelivery period) (Table 3). The extent of these associations also varied by timing of diagnosis. During the delivery hospitalization, residing in the western region compared with the northeastern region was associated with a higher likelihood of SMM (aOR, 1.37; 95% CI, 1.32-1.41); after the delivery hospitalization, residing in the southern region compared with the northeastern region was associated with a higher likelihood of SMM (aOR, 1.29; 95% CI, 1.18-1.39). During the delivery hospitalization, stillbirth delivery was associated with an approximately 4-fold higher likelihood of SMM (aOR, 3.80; 95% CI, 3.53-4.10), and cesarean delivery was associated with an approximately 3-fold higher likelihood of SMM (aOR, 2.89; 95% CI, 2.82-2.95). After the delivery hospitalization, a 2-fold higher likelihood of SMM was observed among women with a stillbirth delivery (aOR, 2.01; 95% CI, 1.60-2.54) or a cesarean delivery (aOR, 2.13; 95% CI, 2.01-2.25).

## Discussion

We found that during delivery hospitalization and within the first 6 weeks after delivery discharge, 14.1% and 15.7% of SMM events among women with commercial insurance and Medicaid, respectively, were first identified after the initial delivery discharge. This finding suggests that focusing on SMM events that occur only during the delivery hospitalization period would omit more than 1 in 7 SMM cases and limit consideration of the importance of outpatient care for maternal health. Most of the de novo SMM cases were identified within the first 2 weeks after delivery discharge.

We also found changes in the most common factors associated with SMM between the delivery hospitalization and the postdelivery discharge period, with infection-associated and cardiovascular-associated factors (eg, sepsis and pulmonary edema or acute heart failure) occurring more often during the postdelivery discharge period compared with the delivery hospitalization period.

Our results suggest that the racial and geographic disparities in SMM during delivery hospitalization, which have been reported previously,^13,22^ also extend to postpartum hospitalization. For example, Black women (in the Medicaid cohort) and women residing in the southern region (in the commercial insurance cohort) had a higher likelihood of experiencing de novo SMM after delivery discharge. In contrast, non-White women (in the Medicaid cohort) and women residing in the northeastern region (in the commercial insurance cohort) had a higher likelihood of experiencing SMM during the delivery hospitalization. These new characterizations of the ways in which postdischarge SMM outcomes are inequitably distributed by race, ethnicity, and geography suggest areas for future research to identify opportunities to prevent SMM. Consistent with the findings of previous studies,^23^ our results indicate that during the delivery hospitalization period, the risk of SMM is higher among women who have stillbirth deliveries compared with those who have live birth deliveries. Such disparity, while somewhat reduced, also persists in the postdelivery discharge period.

The list of factors associated with SMM used in the current study and in many other studies was initially developed using hospital delivery discharge data.^5,6^ Therefore, most previous studies have examined the rates, disparities, distribution, and risk factors associated with SMM during delivery hospitalization. Relatively few studies have assessed newly diagnosed SMM after delivery discharge. Callaghan et al^6^ developed the SMM algorithm and applied it to a national database of hospital discharge records. They reported the rates of SMM during delivery hospitalizations and postpartum hospitalizations separately. Because these data were not linked to follow-up longitudinal data on individual-level admission and discharge outcomes, it was unclear whether the SMM cases identified in the postpartum discharges were new-onset cases.

Using a longitudinally linked database from Massachusetts between 2002 and 2011, Harvey et al^16^ studied the association of SMM during delivery hospitalization with subsequent rehospitalizations within 6 weeks postpartum and 1 year postpartum. They found that SMM was associated with a minimum 2-fold increase in the risk of rehospitalization; however, they did not assess SMM that was diagnosed after delivery hospitalization. Girsen et al^17^ studied the rate, timing, and specific factors associated with SMM at postpartum readmission using linked birth cohort files from California between 2008 and 2012. They reported that approximately 12% of SMM events occurred at postpartum readmission, and approximately one-half of these readmissions occurred within the first week after delivery discharge. Their overall objectives and main results were similar to ours, although there are several differences between their analyses and our study. We used a nationwide commercial insurance claims database and Medicaid claims data from multiple states, so the estimates in our study were derived from a more geographically diverse sample. Our specific aims were also different. We explicitly assessed de novo postdischarge SMM cases, compared them with SMM events observed at delivery hospitalization, and examined associations between maternal characteristics and SMM during different periods.

 Although most SMM cases occurred during the delivery hospitalization, the results of our study indicated that 14.1% and 15.7% of de novo cases occurred in the immediate postpartum period. It is unclear whether SMM first identified after delivery discharge differs in severity from SMM identified during the delivery hospitalization, and the extent to which they are preventable is also unknown. Nevertheless, our results suggest the importance of quality care and follow-up during the postpartum period. Because of the long-lasting consequences of SMM,^16^ efforts to reduce SMM, either at delivery or during the immediate postpartum period, could potentially prevent pregnancy-associated deaths that occur after the immediate postpartum period. As recommended by the American College of Obstetricians and Gynecologists, postpartum care may be considered an ongoing process to meet each woman’s specific needs.^24^ Future studies of SMM may consider expanding the time frame to include new-onset cases observed after the delivery hospitalization because many of these cases may represent a distinct population requiring different interventions to prevent the occurrence of SMM.

### Limitations

This study has several limitations. First, the commercial insurance database component comprised a convenience sample of the US population, and the Medicaid data was obtained from multiple unknown states, which may impact the representativeness of our results regarding de novo SMM rates after delivery discharge and racial and geographic disparities. Second, the data consisted of limited individual characteristics. Race and ethnicity are missing in the commercial insurance data, and geographic information is missing in the Medicaid data. We also did not examine patient comorbidities in the analyses; however, our regression estimates are similar to those that include patient comorbidity factors, such as transient hypertension of pregnancy or preexisting diabetes (eTable 5 in the Supplement). Separate studies using data with more detailed demographic characteristics, such as examinations of state-level linked databases of vital records and hospital discharge data or systematic investigations of the extent to which comorbidities have different associations with SMM occurring during or after delivery, are needed.

Third, a common limitation of administrative databases is that the diagnosis and procedure codes are recorded for billing rather than research purposes. Coding intensity (ie, the mean number of diagnosis or procedure codes reported per claim), general errors, or errors of omission made by medical coders may introduce bias. The intensity of coding has been reported to be associated with the rate of SMM.^25^ Although we were unable to fully ascertain the accuracy of the classifications of delivery and SMM, we used widely adopted and validated algorithms to identify pregnancy outcomes and SMM cases.^18,20^

Fourth, the restriction of our sample to those with continuous insurance enrollment for at least 6 weeks after discharge reduced the sample size by 6% in the commercial insurance cohort and 11% in the Medicaid cohort. The reduced size of the Medicaid cohort is concerning. Depending on the health status of those who disenrolled, our estimates of SMM rates by timing of diagnosis may be biased upward or downward. Nevertheless, the 6-week postdischarge enrollment restriction was a minimum necessary requirement to ensure comparability. It is also notable that the most common factors associated with SMM in the Medicaid cohort are consistent with those observed in the commercial insurance cohort. Fifth, we only examined the timing of SMM diagnosis within the context of a specific delivery. Therefore, we did not consider whether a woman experienced SMM in a previous delivery.

Sixth, the algorithm for SMM was developed for delivery hospitalization. Inaccuracies associated with applying the SMM algorithm to the postdelivery discharge period may have occurred. For instance, the farther away from the delivery date, the less confidence we had about whether an SMM was obstetric-associated. We mitigated this concern by analyzing only the first 6 weeks after delivery rather than a more extended period. This time frame may also be considered a limitation because assessments of pregnancy-associated deaths typically examine the year after delivery.^1,2^ Because we only focused on the first 6 weeks after delivery, we do not know the exact number of de novo SMM cases that occurred outside of delivery hospitalizations, and the total percentage may be higher. Nevertheless, when we extended the postdischarge time frame, we found that most de novo SMM still occurred in the first 2 weeks after delivery discharge.

## Conclusions

Our results highlight the importance of examining SMM that first emerges during the immediate postpartum period. Initiatives such as the State Perinatal Quality Collaboratives program and the Alliance for Innovation on Maternal Health program are actively developing quality improvement projects aimed at reducing maternal morbidity and mortality in the postpartum period. The postpartum care bundles from the Alliance for Innovation on Maternal Health,^26^ for example, provide guidelines to help standardize postpartum care. With increasing focus on maternal health, continued monitoring of SMM can help health care professionals and stakeholders establish priorities and best practices in maternity care. Our research indicated that expanding the focus of SMM assessment to the postdischarge period could provide new insights into the burden of morbidity, and future research in this area may create new opportunities to improve maternity care.

## Notes

Supplement. **eTable 1.** Factors Associated With Severe Maternal Morbidity and Corresponding *ICD-9-CM* Codes **eTable 2.** Number of Women Included and Excluded Because of 2-Month Clean Period Restriction **eTable 3.** *ICD-9-CM* Codes for Stillbirth and Cesarean Deliveries **eTable 4.** Percentage of Women With Specific Factors Associated With Severe Maternal Morbidity in the Commercial Claims and Encounters Database and the Multi-State Medicaid Database, 2010 to 2014 **eTable 5.** Multinomial Logistic Regression Analysis of Severe Maternal Morbidity for Selected Maternal Characteristics by Insurance Type and Timing of Diagnosis with Additional Covariates, 2010 to 2014
