# Error in Abstract Conclusions and Relevance

**DOI:** 10.1001/jamanetworkopen.2021.3451

**Published:** 2021-03-03

In the Original Investigation titled “Assessment of Incidence and Factors Associated With Severe Maternal Morbidity After Delivery Discharge Among Women in the US,”^1^ published February 2, 2021, there was an error in the Abstract Conclusions and Relevance section. The phrase “14.1% of SMM cases in the Medicaid cohort and 15.7% of SMM cases in the commercial insurance cohort” reversed the percentage values. The phrase should have read “15.7% of SMM cases in the Medicaid cohort and 14.1% of SMM cases in the commercial insurance cohort.” This article has been corrected.^1^
