# Supplementary material for: Assessment of Incidence and Factors Associated With Severe Maternal Morbidity After Delivery Discharge Among Women in the US
Source: JAMA Netw Open. 2021 Feb 2;4(2):e2036148. doi: 10.1001/jamanetworkopen.2020.36148 (PMC7856547; doi:10.1001/jamanetworkopen.2020.36148)
Supplement: Supplement. — eTable 1. Factors Associated With Severe Maternal Morbidity and Corresponding ICD-9-CM Codes eTable 2. Number of Women Included and Excluded Because of 2-Month Clean Period Restriction eTable 3. ICD-9-CM Codes for Stillbirth and Cesarean Deliveries eTable 4. Percentage of Women With Specific Factors Associated With Severe Maternal Morbidity in the Commercial Claims and Encounters Database and the Multi-State Medicaid Database, 2010 to 2014 eTable 5. Multinomial Logistic Regression Analysis of Severe Maternal Morbidity for Selected Maternal Characteristics by Insurance Type and Timing of Diagnosis with Additional Covariates, 2010 to 2014 [file jamanetwopen-e2036148-s001.pdf]

Supplementary Online Content

Chen J, Cox S, Kuklina EV, Ferre C, Barfield W, Li R. Assessment of incidence and factors associated with severe maternal morbidity after delivery discharge among women in the US. *JAMA Netw Open*. 2021;4(2):e2036148. doi:10.1001/jamanetworkopen.2020.36148

eTable 1. Factors Associated With Severe Maternal Morbidity and Corresponding *ICD-9-CM* Codes

eTable 2. Number of Women Included and Excluded Because of 2-Month Clean Period Restriction

eTable 3. *ICD-9-CM* Codes for Stillbirth and Cesarean Deliveries

eTable 4. Percentage of Women With Specific Factors Associated With Severe Maternal Morbidity in the Commercial Claims and Encounters Database and the Multi-State Medicaid Database, 2010 to 2014

eTable 5. Multinomial Logistic Regression Analysis of Severe Maternal Morbidity for Selected Maternal Characteristics by Insurance Type and Timing of Diagnosis With Additional Covariates, 2010 to 2014

This supplementary material has been provided by the authors to give readers additional information about their work.

© 2021 Chen J et al. *JAMA Network Open*.

eTable 1. Factors Associated With Severe Maternal Morbidity and Corresponding *ICD-9-CM* Codes

Severe Maternal Morbidity Indicators	DX or PR	ICD-9-CM
1. Acute myocardial infarction	DX	410.xx
2. Aneurysm	DX	441.xx
3. Acute renal failure	DX	584.5, 584.6, 584.7, 584.8, 584.9, 669.3x
4. Adult respiratory distress syndrome	DX	518.5x, 518.81, 518.82, 518.84, 799.1
5. Amniotic fluid embolism	DX	673.1x
6. Cardiac arrest/ventricular fibrillation	DX	427.41, 427.42, 427.5
7. Conversion of cardiac rhythm	PR	99.6x
8. Disseminated intravascular coagulation	DX	286.6, 286.9, 666.3x
9. Eclampsia	DX	642.6x
10. Heart failure/arrest during surgery or procedure	DX	669.4x, 997.1
11. Puerperal cerebrovascular disorders	DX	430.xx, 431.xx, 432.xx, 433.xx, 434.xx, 436.xx, 437.xx, 671.5x, 674.0x, 997.02
12. Pulmonary edema and acute heart failure	DX	518.4, 428.1, 428.0, 428.21, 428.23, 428.31, 428.33, 428.41, 428.43
13. Severe anesthesia complications	DX	668.0x, 668.1x, 668.2x
14. Sepsis	DX	038.xx, 995.91, 995.92, 670.2x
15. Shock	DX	669.1x, 785.5x, 995.0, 995.4, 998.0x

© 2021 Chen J et al. *JAMA Network Open*.

16. Sickle cell disease with crisis	DX	282.42, 282.62, 282.64, 282.69
17. Air and thrombotic embolism	DX	415.1x, 673.0x, 673.2x, 673.3x, 673.8x
18. Blood products transfusion	PR	99.0x
19. Hysterectomy	PR	68.3x-68.9x
20. Temporary tracheostomy	PR	31.1
21. Ventilation	PR	93.90, 96.01, 96.02, 96.03, 96.05

Abbreviations: DX, diagnosis code; PR, procedure code; ICD-9-CD, the International Classification of Disease, 9th Revision, Clinical Modification. Source: https://www.cdc.gov/reproductivehealth/maternalinfanthealth/smm/severe-morbidity-ICD.htm

© 2021 Chen J et al. *JAMA Network Open*.

eTable 2. Number of Women Included and Excluded Because of 2-Month Clean Period Restriction

	Any SMM indicator during the two months before delivery	Any SMM indicator during the delivery hospitalization	Any SMM indicator after the delivery hospitalization	*N*
Panel A: Medicaid-insured				
Included categories (N = 809,377*)*				
no SMM (during and after delivery)	×	×	×	788,528
any SMM during delivery	×	✓	×	17,180
any SMM during delivery	×	✓	✓	404
any de novo post-discharge SMM	×	×	✓	3,265
Excluded categories (N = 1,043)				
	✓	×	×	724
	✓	×	✓	29
	✓	✓	×	240
	✓	✓	✓	50
Panel B: Commercially-insured				
Included categories (N = 1,857,948*)*				
no SMM (during and after delivery)	×	×	×	1,820,594
any SMM during delivery	×	✓	×	31,574
any SMM during delivery	×	✓	✓	505
any de novo post-discharge SMM	×	×	✓	5,275
Excluded categories (N = 1,186)				
	✓	×	×	898
	✓	×	✓	19
	✓	✓	×	250
	✓	✓	✓	19

Notes: "✓" ("×") indicates the presence (absence) of any SMM indicator for the corresponding period.

© 2021 Chen J et al. *JAMA Network Open*.

eTable 3. *ICD-9-CM* Codes for Stillbirth and Cesarean Deliveries

Indicators	DX or PR	ICD-9-CM
Any Stillbirth	DX	651.31, 651.41, 651.51, 656.4X, V27.1, V27.3, V27.4, V27.6, V27.7, V32.0, V32.00, V32.01, V35.0, V35.00, V35.01, V36.0, V36.00, V36.01
Cesarean Delivery	DX	669.70, 669.71
	PR	740, 741, 742, 744, 7499

Abbreviations: DX, diagnosis code; PR, procedure code; ICD-9-CD, the International Classification of Disease, 9th Revision, Clinical Modification.

© 2021 Chen J et al. *JAMA Network Open*.

eTable 4. Percentage of Women With Specific Factors Associated With Severe Maternal Morbidity in the Commercial Claims and Encounters Database and the Multi-State Medicaid Database, 2010 to 2014

	SMM with blood transfusion		SMM without blood transfusion	
Panel A: Medicaid-insured				
Indicator: n (pct.) [rank]	During delivery (N = 17,584)	After delivery (N = 3,265)	During delivery (N = 6,914)	After delivery (N = 2,520)
Blood products transfusion	10,670 (60.7) [1]	745 (22.8) [2]	--- (---) [---]	--- (---) [---]
Disseminated intravascular coagulation	2,514 (14.3) [2]	116 (3.6) [9]	2,514 (36.4) [1]	116 (4.6) [8]
Heart failure/arrest during surgery or procedure	1,208 (6.9) [3]	103 (3.2) [10]	1,208 (17.5) [2]	103 (4.1) [9]
Eclampsia	1,181 (6.7) [4]	346 (10.6) [6]	1,181 (17.1) [3]	346 (13.7) [5]
Adult respiratory distress syndrome	1,015 (5.8) [5]	449 (13.8) [4]	1,015 (14.7) [4]	449 (17.8) [3]
Pulmonary edema / Acute heart failure	739 (4.2) [6]	907 (27.8) [1]	739 (10.7) [5]	907 (36.0) [1]
Acute renal failure	722 (4.1) [7]	249 (7.6) [8]	722 (10.4) [6]	249 (9.9) [7]
Hysterectomy	597 (3.4) [8]	53 (1.6) [12]	597 (8.6) [7]	53 (2.1) [11]
Sepsis	472 (2.7) [9]	631 (19.3) [3]	472 (6.8) [8]	631 (25.0) [2]
Shock	345 (2.0) [10]	74 (2.3) [11]	345 (5.0) [9]	74 (2.9) [10]
Puerperal cerebrovascular disorders	340 (1.9) [11]	265 (8.1) [7]	340 (4.9) [10]	265 (10.5) [6]
Air and thrombotic embolism	297 (1.7) [12]	387 (11.9) [5]	297 (4.3) [11]	387 (15.4) [4]
Ventilation	145 (0.8) [13]	43 (1.3) [13]	145 (2.1) [12]	43 (1.7) [12]
Severe anesthesia complications	106 (0.6) [14]	21 (0.6) [15]	106 (1.5) [13]	21 (0.8) [14]
Sickle cell disease with crisis	101 (0.6) [15]	9 (0.3) [18]	101 (1.5) [14]	9 (0.4) [17]
Cardiac arrest/ventricular fibrillation	62 (0.4) [16]	17 (0.5) [16]	62 (0.9) [15]	17 (0.7) [15]
Conversion of cardiac rhythm	45 (0.3) [17]	7 (0.2) [19]	45 (0.7) [16]	7 (0.3) [18]
Acute myocardial infarction	43 (0.2) [18]	41 (1.3) [14]	43 (0.6) [17]	41 (1.6) [13]
Aneurysm	18 (0.1) [19]	4 (0.1) [20]	18 (0.3) [18]	4 (0.2) [19]
Temporary tracheostomy	17 (0.1) [20]	13 (0.4) [17]	17 (0.2) [19]	13 (0.5) [16]
Amniotic fluid embolism	5 (0.0) [21]	0 (0.0) [21]	5 (0.1) [20]	0 (0.0) [20]
Panel B: Commercially-insured				
Indicator: n (pct.) [rank]	During delivery (N = 32,079)	After delivery (N = 5,275)	During delivery (N = 15,753)	After delivery (N = 3,799)
Blood products transfusion	16,326 (50.9) [1]	1,476 (28.0) [1]	--- (---) [---]	--- (---) [---]

© 2021 Chen J et al. *JAMA Network Open*.

Disseminated intravascular coagulation	5,884 (18.3) [2]	210 (4.0) [9]	5,884 (37.4) [1]	210 (5.5) [8]
Heart failure/arrest during surgery or procedure	2,096 (6.5) [3]	186 (3.5) [10]	2,096 (13.3) [2]	186 (4.9) [9]
Eclampsia	1,955 (6.1) [4]	404 (7.7) [7]	1,955 (12.4) [3]	404 (10.6) [6]
Adult respiratory distress syndrome	1,583 (4.9) [5]	495 (9.4) [5]	1,583 (10.0) [4]	495 (13.0) [4]
Pulmonary edema / Acute heart failure	1,053 (3.3) [9]	1,225 (23.2) [2]	1,053 (6.7) [8]	1,225 (32.2) [1]
Acute renal failure	1,100 (3.4) [8]	245 (4.6) [8]	1,100 (7.0) [7]	245 (6.4) [7]
Hysterectomy	1,289 (4.0) [7]	152 (2.9) [11]	1,289 (8.2) [6]	152 (4.0) [10]
Sepsis	886 (2.8) [10]	918 (17.4) [3]	886 (5.6) [9]	918 (24.2) [2]
Shock	747 (2.3) [12]	140 (2.7) [12]	747 (4.7) [11]	140 (3.7) [11]
Puerperal cerebrovascular disorders	816 (2.5) [11]	491 (9.3) [6]	816 (5.2) [10]	491 (12.9) [5]
Air and thrombotic embolism	545 (1.7) [13]	591 (11.2) [4]	545 (3.5) [12]	591 (15.6) [3]
Ventilation	1,459 (4.5) [6]	23 (0.4) [15.5]	1,459 (9.3) [5]	23 (0.6) [14.5]
Severe anesthesia complications	219 (0.7) [14]	23 (0.4) [15.5]	219 (1.4) [13]	23 (0.6) [14.5]
Sickle cell disease with crisis	69 (0.2) [18]	7 (0.1) [20]	69 (0.4) [17]	7 (0.2) [19]
Cardiac arrest/ventricular fibrillation	99 (0.3) [16]	24 (0.5) [14]	99 (0.6) [15]	24 (0.6) [13]
Conversion of cardiac rhythm	136 (0.4) [15]	13 (0.2) [18]	136 (0.9) [14]	13 (0.3) [17]
Acute myocardial infarction	78 (0.2) [17]	99 (1.9) [13]	78 (0.5) [16]	99 (2.6) [12]
Aneurysm	53 (0.2) [19]	10 (0.2) [19]	53 (0.3) [18]	10 (0.3) [18]
Temporary tracheostomy	27 (0.1) [20]	17 (0.3) [17]	27 (0.2) [19]	17 (0.4) [16]

Notes: Each woman may have more than one indicator.

© 2021 Chen J et al. *JAMA Network Open*.

eTable 5. Multinomial Logistic Regression Analysis of Severe Maternal Morbidity for Selected Maternal Characteristics by Insurance Type and Timing of Diagnosis with Additional Covariates, 2010 to 2014[a]

Characteristic by insurance type	aOR (95% CI)		*P value*[b]
	Any SMM indicator during delivery hospitalization vs. none	Any de novo SMM indicator during postpartum hospitalization vs. none	
Medicaid (*n* = 809,377)[c]			
Age group (years)			
15-24	1.0 [Reference]	1.0 [Reference]	NA
25-34	0.88 [0.85,0.91]	1.29 [1.19,1.39]	<.001
35-44	1.05 [1.00,1.11]	1.66 [1.48,1.86]	<.001
Race/ethnicity			
White	1.0 [Reference]	1.0 [Reference]	NA
Black	1.46 [1.41,1.51]	1.62 [1.50,1.74]	.015
Hispanic	1.49 [1.40,1.60]	0.88 [0.73,1.06]	<.001
Other	1.40 [1.34,1.48]	0.97 [0.85,1.10]	<.001
Fetal outcome			
No stillbirth	1.0 [Reference]	1.0 [Reference]	NA
Any stillbirth	4.38 [4.03,4.77]	1.61 [1.23,2.12]	<.001
Delivery method			
Vaginal delivery	1.00 [Reference]	1.00 [Reference]	NA
Cesarean delivery	3.66 [3.54,3.77]	1.96 [1.83,2.10]	<.001
Comorbidity			
Transient hypertension of pregnancy	1.43 [1.36,1.51]	1.51 [1.33,1.70]	.465
Multiple gestation	1.58 [1.46,1.72]	1.50 [1.22,1.85]	.643
Preexisting diabetes mellitus	1.13 [1.03,1.23]	1.54 [1.28,1.85]	.002
Preexisting hypertension	2.04 [1.94,2.15]	1.85 [1.64,2.09]	.139
Commercially (*n* = 1,857,948)[d]			
Age group (years)			
15-24	1.0 [Reference]	1.0 [Reference]	NA
25-34	0.87 [0.84,0.90]	0.85 [0.78,0.92]	.554

© 2021 Chen J et al. *JAMA Network Open*.

35-44	0.99 [0.95,1.03]	1.12 [1.03,1.23]	.011
Census region [e]			
Northeast	1.0 [Reference]	1.0 [Reference]	NA
North central	0.86 [0.83,0.90]	1.09 [0.99,1.19]	<.001
South	0.80 [0.78,0.83]	1.23 [1.13,1.34]	<.001
West	1.37 [1.32,1.41]	1.06 [0.96,1.16]	<.001
Metropolitan statistical area [e]			
MSA	1.0 [Reference]	1.0 [Reference]	NA
Non-MSA	1.03 [0.99,1.06]	1.04 [0.96,1.13]	.691
Fetal outcome			
No stillbirth	1.0 [Reference]	1.0 [Reference]	NA
Any stillbirth	3.49 [3.23,3.76]	1.90 [1.50,2.39]	<.001
Delivery method			
Vaginal delivery	1.0 [Reference]	1.0 [Reference]	
Cesarean delivery	2.71 [2.64,2.77]	1.98 [1.87,2.09]	<.001
Comorbidity			
Transient hypertension of pregnancy	1.32 [1.26,1.38]	1.60 [1.45,1.76]	<.001
Multiple gestation	1.92 [1.82,2.03]	1.48 [1.26,1.73]	.002
Preexisting diabetes mellitus	1.26 [1.17,1.35]	1.60 [1.36,1.87]	.006
Preexisting hypertension	2.10 [2.01,2.19]	2.24 [2.03,2.47]	.237

Abbreviations: aOR, adjusted odds ratio; MSA, metropolitan statistical area (as defined by the US Office of Management and Budget); NA, not applicable; SMM, severe maternal morbidity.

[a]Estimates are exponentiated coefficients from a single multinomial 4 regression analysis for each insurance category. The dependent variable is a discrete variable with 3 distinct values: (1) no SMM during the delivery hospitalization and the postdelivery period (reference group), (2) at least 1 factor associated with SMM during the delivery hospitalization, and (3) any factor associated with de novo SMM after delivery discharge (defined as SMM that was first diagnosed in the inpatient setting during the 6 weeks [or 42 days] after discharge from the delivery hospitalization, conditional on no factor associated with SMM being identified during delivery).

[b]P values were obtained from $\chi 2$ tests of the equality of coefficients.

[c]Data were obtained from the 2010 to 2014 IBM MarketScan Multi-State Medicaid database.

[d]Data were obtained from the 2010 to 2014 IBM MarketScan Commercial Claims and Encounters database.

[e]Census region and MSA status are categorical variables with a missing/unknown category. Estimates for the missing/unknown category are not included in the table.

© 2021 Chen J et al. *JAMA Network Open*.
